# Identification of a Specific Vimentin Isoform That Induces an Antibody Response in Pancreatic Cancer

**Published:** 2007-02-07

**Authors:** Su-Hyung Hong, David E. Misek, Hong Wang, Eric Puravs, Robert Hinderer, Thomas J. Giordano, Joel K. Greenson, Dean E. Brenner, Diane M. Simeone, Craig D. Logsdon, Samir M. Hanash

**Affiliations:** Departments of Pediatrics (SHH, DEM, HW, EP, RH, SMH), Pathology (TJG and JKG), Physiology (CDL), Surgery (DMS) and Internal Medicine (DEB), University of Michigan Medical School, 1150 W. Medical Center Drive, Ann Arbor, Michigan 48109 and the Department of Dental Microbiology (SHH), School of Dentistry, Kyungpook National University, 101 Dongin-Dong, Jung-Gu, Daegu, 700-422, South Korea

## Abstract

Pancreatic cancer has a poor prognosis, in part due to lack of early detection. The identification of circulating tumor antigens or their related autoantibodies provides a means for early cancer diagnosis. We have used a proteomic approach to identify proteins that commonly induce a humoral response in pancreatic cancer. Proteins from a pancreatic adenocarcinoma cell line (Panc-1) were subjected to two-dimensional PAGE, followed by Western blot analysis in which individual sera were tested for autoantibodies. Sera from 36 newly diagnosed patients with pancreatic cancer, 18 patients with chronic pancreatitis and 15 healthy subjects were analyzed. Autoantibodies were detected against a protein identified by mass spectrometry as vimentin, in sera from 16/36 patients with pancreatic cancer (44.4%). Only one of 18 chronic pancreatitis patients and none of the healthy controls exhibited reactivity against this vimentin isoform. Interestingly, none of several other isoforms of vimentin detectable in 2-D gels exhibited reactivity with patient sera. Vimentin protein expression levels were investigated by comparing the integrated intensity of spots visualized in 2-D PAGE gels of various cancers. Pancreatic tumor tissues showed greater than a 3-fold higher expression of total vimentin protein than did the lung, colon, and ovarian tumors that were analyzed. The specific antigenic isoform was found at 5–10 fold higher levels. The detection of autoantibodies to this specific isoform of vimentin may have utility for the early diagnosis of pancreatic cancer.

## Introduction

There is substantial evidence for a humoral immune response to cancer in humans, demonstrated by the identification of autoantibodies to a number of intracellular and surface antigens in serum from patients with different tumor types (1–3). A tumor-specific humoral immune response directed against oncoproteins (4–5), or mutated proteins such as p53 (6) or other aberrantly expressed proteins have been previously described. It is currently largely unknown whether the occurrence of such antibodies is beneficial. However, knowledge of potential tumor antigens that may evoke tumor-specific immune responses may have relevance to the development of effective strategies for cancer screening and diagnosis.

Pancreatic cancer has the worst prognosis among cancers, with a 5-year survival rate of <3%, accounting for the fourth largest number of cancer deaths in the United States (7). The poor prognosis for pancreatic cancer is, in part, due to lack of early detection methods. Currently, there is a paucity of pancreatic cancer markers for early detection and for the diagnosis of pancreatic cancer.

Autoimmunity in pancreatic cancer has been demonstrated against several proteins, including MUC1 (8–9), p53 (9), calreticulin (13) and Rad51 (10) proteins. MUC1 is a transmembrane glycoprotein involved in cell-cell and cell-extracellular matrix interactions, and MUC1 autoantibodies have been observed in sera from patients with a variety of different tumors (11). The presence of MUC1 IgG autoantibodies has been shown to be associated with a favorable prognosis (8–9). The recombination factor Rad51 is highly expressed in pancreatic adenocarcinoma (10), and Rad51 autoantibodies have been observed in 7% of patients with pancreatic cancer.

A large number of autoantibodies have been identified in various tumor types, but in most cases they occur in a small percentage of patient’s sera. Therefore, they are not effective individually for the early detection of cancer. Thus, the development of panels of such autoantibodies directed against a variety of tumor antigens may be effective (12).

We have implemented a proteomic approach for the identification of tumor antigens that elicit a humoral response in pancreatic cancer (13). In this study we have utilized the pancreatic cancer cell line Panc-1 as the source of tumor cell proteins for antigen identification. We have utilized 2-D PAGE to separate protein constituents, followed by their transfer onto PVDF membranes. Sera from cancer patients and from controls were screened individually by Western blot analysis for antibodies that reacted against the resolved proteins. Mass spectrometric analysis was used for protein identification. Within, we report the identification of a vimentin isoform as an antigen that elicits a humoral immune response in pancreatic cancer.

## Materials and Methods

### Materials

All cell culture reagents, including Dulbecco’s modified Eagle’s medium (DMEM, containing L-glutamine, sodium pyruvate and pyridoxine hydrochloride), Dulbecco’s phosphate buffered saline (PBS), fetal calf serum and penicillin/streptomycin were obtained from Invitrogen (Carlsbad, CA). The mouse monoclonal anti-vimentin antibody (Clone V9) was purchased from Lab Vision Corp. (Fremont, CA). The horseradish peroxidase-conjugated sheep anti-human IgG and the ECL (Enhanced Chemiluminescence) kits were obtained from Amersham (Piscataway, NJ). The Immobilon-P PVDF (polyvinylidene fluoride) membranes were purchased from Millipore Corp. (Bedford, MA). The acrylamide used in the first dimension electrophoresis, urea, ammonium persulfate and PDA (piperazine diacrylamide) were all purchased from BioRad (Rockville Center, NY). The acrylamide used in the second dimension electrophoresis was purchased from Serva (Crescent Chemical, Hauppauge, NY) and the carrier ampholytes (pH 4 to 8) and NP-40 were both purchased from Gallard/Schlessinger (Carle Place, NY). All other reagents and chemicals were obtained from either Fisher or Sigma and were of the highest purity available.

### Sera, tumor tissues and cell lines

Serum and tumor tissue was obtained at the time of diagnosis following informed consent using IRB-approved guidelines. A total of 36 serum samples were obtained from patients with a confirmed diagnosis of pancreatic adenocarcinoma who were seen in the Multidisciplinary Pancreatic Tumor Clinic at the University of Michigan Comprehensive Cancer Center. Sera from the pancreatic cancer patients were randomly selected from a clinic population that sees, on average, at the time of initial diagnosis, 15% of pancreatic adenocarcinoma patients presenting with early stage (i.e. stage 1/2) disease and 85% presenting with advanced stage (i.e. stage 3/4). Inclusion criteria for the study included patients with a confirmed diagnosis of pancreatic cancer, the ability to provide written, informed consent, and the ability to provide 40 ml of blood. Exclusion criteria included inability to provide informed consent, patients actively undergoing chemotherapy or radiation therapy for pancreatic cancer, and patients with other malignancies diagnosed or treated within the last 5 years. Sera were also obtained from 18 patients with chronic pancreatitis who were seen in the Gastroenterology Clinic at University of Michigan Medical Center and at the Catholic University of Daegu, in Daegu, South Korea, and from 15 control healthy individuals collected at University of Michigan under the auspices of the Early Detection Research Network (EDRN). The mean age of the tumor group was 65.4 years (range 54–74 years) and from the chronic pancreatitis group was 54 years (range 45–65). The sera from the normal subject group was age and sex-matched to the tumor group. All of the chronic pancreatitis sera were collected in an elective setting in the clinic in the absence of an acute flare. All sera were processed using identical procedures. The samples were permitted to sit at room temperature for a minimum of 30 minutes (and a maximum of 60 minutes) to allow the clot to form in the red top tubes, and then centrifuged at 1,300 × g at 4°C for 20 minutes. The serum was removed, transferred to a polypropylene, capped tube in 1 ml aliquots, and frozen. The frozen samples were stored at −70°C until assayed. All serum samples were labeled with a unique identifier to protect the confidentiality of the patient. None of the samples were thawed more than twice before analysis. The human cancer cell lines used in this study were all individually cultured in Dulbecco’s modified Eagle medium supplemented with 10% fetal bovine serum, 100 units/ml penicillin and 100 units/ml streptomycin (Invitrogen, Carlsbad, CA).

### 2-D PAGE and Western blot analysis

After excision, the tumor tissue was immediately frozen at −80°C. An aliquot was lysed in solubilization buffer (8 M urea (Bio-Rad), 2% Nonidet P-40, 2% carrier ampholytes, pH 4–8 (Gallard/Schlessinger), 2% β-mercaptoethanol, and 10 mM PMSF), then stored at −80°C until use. Cultured Panc-1 pancreatic adenocarcinoma cells were harvested in 300 μl of solubilization buffer by using a cell scraper and stored at −80°C until use. Proteins derived from the extracts of either cultured cells or solid tumors were separated into two dimensions as described previously (14). Briefly, solubilized proteins were applied onto isoelectric focusing gels. Isoelectric focusing was performed using pH 4 to 8 carrier ampholytes at 700 V for 16 h, followed by 1000V for an additional 2 h. The first-dimension gel was loaded onto the second-dimension gel, after equilibration in 125 mM Tris, pH 6.8, 10% glycerol, 2% SDS, 1% dithiothreitol, and bromophenol blue. For the second-dimension separation, a gradient of 11 to 14 % acrylamide (Crescent Chemical) was used. Proteins were transferred to an Immobilon-P PVDF membrane (Millipore) or visualized by silver staining of the gels.

### Western blotting

After transfer, membranes were incubated with a blocking buffer consisting of 10 mM Tris-HCl (pH 7.5), 50 mM NaCl, 1.8% nonfat dry milk, and 0.01% Tween 20 for 2 h. The membranes were incubated for 1 h at room temperature with serum obtained from either patients or healthy individuals as a source of primary antibody at a 1:100 dilution. Following three washes with washing buffer (Tris-buffered saline containing 0.01% Tween 20), the membranes were incubated with horseradish peroxidase-conjugated anti-human (Amersham) IgG antibodies at a dilution of 1:1000 for 1 h at room temperature. Immunodetection was accomplished by ECL (Enhanced Chemiluminescence (Amersham)) followed by autoradiography on Hyperfilm MP (Amersham).

### Vimentin detection by western blotting

A mouse anti-vimentin (Clone V9) monoclonal antibody (Lab Vision Corporation, Fremont, CA) was used at 1:100 dilution for western blotting and was processed as for incubations with patient sera, with a horseradish peroxidase-conjugated anti-mouse IgG (Amersham) as the secondary antibody.

### In-gel enzyme digestion and mass spectrometry

For protein identification by mass spectrometry, 2-D gels were stained by a modified silver staining method, and excised proteins were destained for 5 min in 15mM potassium ferricyanide and 50 mM sodium thiosulfate as described (15). Following three washes with water, the gel pieces were dehydrated in 100% acetonitrile for 5 min, and then dried. Digestion was performed by addition of 100 ng of trypsin (Promega, Madison, WI) in 200 nM ammonium bicarbonate. Following enzymatic digestion overnight at 37°C, the peptides were extracted twice with 50 μl of 60% acetonitrile/1% trifluoroacetic acid. Following removal of acetonitrile by centrifugation in a vacuum centrifuge, the peptides were concentrated by using pipette tips C18 (Millipore) and identified by nanoflow capillary liquid chromatography coupled with electrospray quadrupole-time of flight tandem mass spectrometry (ESI Q-TOF MS/MS) in the Q-TOF *micro* (MicroMass, Manchester, U.K.). The acquired spectra were processed and searched against a non-redundant Swiss-Prot protein sequence database using protein-Lynx Global Server (www.micromass.co.uk).

## Results

### Autoantibodies to pancreatic tumor proteins in sera from patients with pancreatic cancer

**Figure 1:**
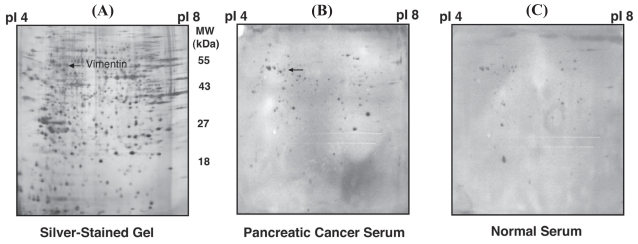
(**A**) A silver-stained image of the Panc-1 pancreatic tumor cell line, (**B**) as compared with a Western blot of the Panc-1 cell line with serum from a patient with pancreatic cancer, and (**C**) normal serum.

Panc-1 pancreatic tumor cell line proteins were resolved by 2-D PAGE, then transferred onto Immobilon-P PVDF membranes. Sera obtained from 36 newly diagnosed patients with pancreatic cancer, from 18 patients with chronic pancreatitis and from 15 healthy donors were screened individually for the presence of antibodies to Panc-1 pancreatic tumor cell line proteins (Table 1). Each membrane was treated with one serum sample as the primary antibody and with sheep anti-human IgG as the secondary antibody. In general, most pancreatic patient sera reacted against multiple proteins (Fig. 1). Some of the reactive proteins also reacted with control sera and thus were considered to represent nonspecific reactivity. The reactive proteins most commonly observed with pancreatic cancer patient sera, but not with controls included one protein with an estimated molecular mass of 55 kDa and a pI of 5. This protein showed reactivity with sera from 16 of 36 patients with pancreatic cancer (44.4%), with 1/18 sera (5.6%, p = 0.003 (one-sided Fisher’s exact test)) from chronic pancreatitis patients and none of 15 (0%, p = 0.001 (one-sided Fisher’s exact test)) sera from healthy donors (Table 1). Combining the chronic pancreatitis and healthy controls into a single group gave p = 4 × 10 − 5 (one-sided Fisher’s exact test) in comparison to cancer.

### Identification of the reactive protein as vimentin

**Figure 2:**
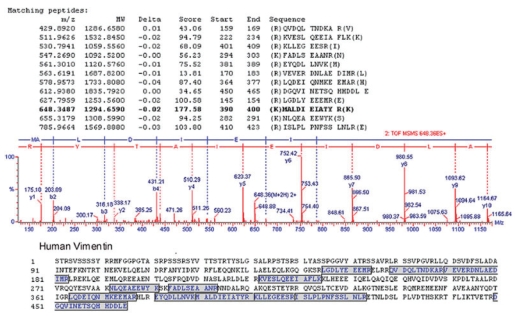
Tandem mass spectrometry identification of vimentin. The MS/MS spectrum of vimentin (obtained after trypsin digestion) is shown by analysis with ESI-Q-TOF, coupled with nanoflow capillary high-performance liquid chromatography. The precursor ion shown in the figure is m/z 648.3487, and resultant peaks were searched against the non-redundant SwissProt protein sequence database using the ProteinLynx global server. A total of twelve tryptic peptides, as shown, matched the vimentin protein.

**Figure 3:**
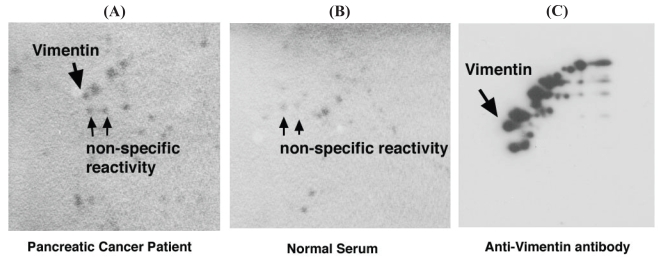
Western blot analysis of vimentin with (**A**) sera from a pancreatic cancer patient, (**B**) from healthy individual, and (**C**) a monoclonal anti-vimentin antibody.

The protein spot of interest was extracted from the gels following 2-D PAGE and silver staining, digested with trypsin and the resulting peptides were analyzed by ESI Q-TOF Tandem MS spectrometry. The acquired spectra were processed and searched against a non-redundant SwissProt protein sequence database using proteinLynx Global Server (www.micromass.co.uk). The sequence of 12 tryptic peptides matched the predicted sequence of vimentin (MW 53.5 kDa. pI 5.1) (Fig. 2). The identity of this protein was confirmed with 2-D Western blotting using Panc-1 whole-cell extracts and anti-vimentin mouse monoclonal antibody (Fig. 3). Interestingly, although a number of vimentin isoforms were detected by monoclonal anti-vimentin antibody from Panc-1 whole cell lysates, only one vimentin isoform showed reactivity specifically with pancreatic cancer patient sera. We hypothesized that perhaps specific protease cleavage of intact vimentin resulted in the observed antigenicity. To this end, each of the three lowest vimentin isoforms which were reactive with patients serum (Fig. 3) were excised from modified silver-stained gels (the uppermost isoform was not visualized in the silver-stained gels), then subjected to analysis by tandem mass spectrometry in order to search for vimentin-specific tryptic peptides. We found multiple peptide coverage (7–13 peptides for each isoform), spanning residues 78–400 for each of the three isoforms (data not shown). Thus, as the same peptide coverage was identified for each of the isoforms, we were unable to find evidence of protease cleavage leading to antigenicity of the specific vimentin isoform that was specifically reactive with pancreatic cancer patient’s serum. Moreover, although some peptide modifications were found, such as deamination, methylation, phosphorylation and hydroxylation, none were specific to the vimentin isoform that showed reactivity specifically with pancreatic cancer patient’s serum (data not shown).

### Role of glycosylation in vimentin antigenicity

We sought to determine whether aberrant vimentin glycosylation contributed to immunogenicity. Solubilized proteins from the Panc-1 cell line were subjected to N-deglycosylation by a combination of Endoglycosidase F, Endo-α-N-acetylgalactosaminidase, and α-2–3, 6, 8, 9-Neuraminidase. The resulting products were separated by SDS electrophoresis and analyzed by Western blotting. Although the deglycosylated positive control revealed a demonstrable mobility shift by SDS-PAGE, the deglycosylating enzyme treatment did not result in any mobility shifts of vimentin. Thus, Endoglycosidase F- sensitive glycosylation does not appear to be playing a role in the observed immunogenicity of the vimentin isoform (data not shown).

### Analysis of vimentin expression by 2-D PAGE

**Figure 4:**
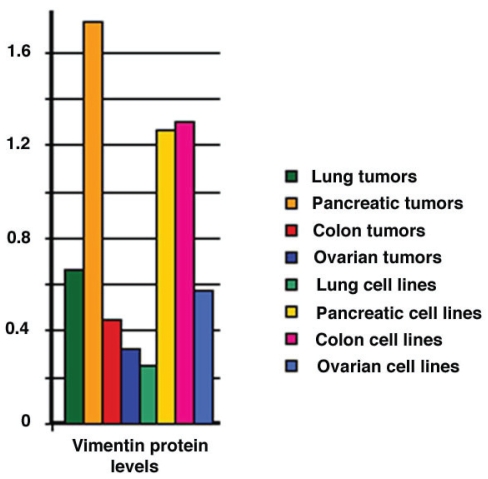
Vimentin protein levels measured in human tumors and tumor cell lines. 2-D gels were prepared using solubilized proteins from a variety of human tumors and tumor cell lines, as described in “Materials and Methods”. Background corrected integrated intensity (volume) was measured (Visage software, Genomic Solutions, Ann Arbor, MI) for total vimentin. Bars represents the average intensities for 38 lung tumors, 6 pancreatic tumors, 7 colon tumors, 25 ovarian tumors, 4 lung cell lines, 6 pancreatic cell lines, 9 colon cell lines and 33 ovarian cell lines.

We hypothesized that there might be changes in the expression level of the single minor antigenic isoform of vimentin that could lead to antigenicity in pancreatic cancer. Using 2-D PAGE, we examined the expression of the different vimentin isoforms in a variety of tissues and tumor types. The major vimentin isoforms were present in the different cell lines examined, including 6 pancreatic tumor cell lines, 4 lung tumor cell lines, 9 colon tumor cell lines and 33 ovarian tumor cell lines, at similar expression levels. A similar pattern of expression was also observed in 6 pancreatic tumors, 38 lung tumors, 7 colon tumors and 25 ovarian tumors (Fig. 4), suggesting that the major vimentin isoforms were ubiquitously expressed. However, the minor isoform that was found to be antigenic was specific to just a subset of the tumor cell lines. We analyzed (by Western blot) different tumor cell lines for the specific isoform of vimentin that was immunogenic in pancreatic tumor patients. These cell lines were derived from 4 lung tumors (3 adenocarcinoma and 1 small cell), 2 pancreatic tumors, 2 breast tumors, 7 colon tumors, 1 neuroblastoma and 1 testes. Interestingly, the isoform of vimentin was only found in 4 of the cell lines analyzed (in the Panc-1 pancreatic, in the H23 and A549 lung adenocarcinoma, and in the Hs1 testes tumor cell lines), thus suggesting that expression of the antigenic isoform was cell line-specific. Additionally, we explored whether the antigenic isoform was overexpressed in a variety of different adenocarcinomas, including 18 colon, 16 ovarian, 14 lung, 10 esophageal and pancreatic (4 tumor and 4 normal tissue). The integrated intensity measurement of the antigenic isoform in each tumor type was directly compared to that of a neighboring non-antigenic vimentin isoform that is ubiquitously expressed in all tumor types examined. Interestingly, the antigenic isoform of vimentin was expressed at 5–10 fold higher levels relative to the ubiquitous isoform in pancreatic tumors compared to other tumor types, and was expressed at approximately 50% higher levels than that found in normal pancreas (Table 2).

## Discussion

We have implemented a proteomics-based approach to identify proteins that elicit a humoral response in pancreatic cancer patients. This approach allows screening by Western blot analysis of patient sera for antibodies that react against separated tumor cell proteins. This study was focused on a search for autoantibodies to pancreatic tumor proteins present in the Panc-1 cancer cell line. We have shown that a humoral response directed against a single isoform of vimentin occurred in 44.4% patients with pancreatic cancer. One out of 18 (5.6%) chronic pancreatitis patients and none of the noncancer controls exhibited reactivity against the antigenic vimentin isoform.

Intermediate filaments are one of the three major cytoskeleton networks in higher eukaryotic cells. These filaments consist of a number of different members, including vimentin and the cytokeratin proteins. Different types of intermediate filament protein genes are expressed depending on the tissue type. Little is known about the function of intermediate filaments in normal cells, although it is believed that they provide cellular integrity and resistance against mechanical stresses (16). Their tissue-specific expression in normal cells and differential expression/assembly in cancer is of great pathologic value in tumor diagnostics. Expression of vimentin has been postulated to play a role in invasiveness and metastasis in cervical carcinoma (17). However, Heatley et al. have shown that vimentin expression could not differentiate between benign and invasive breast lesions, although its expression was correlated with tumor grade and decreased survival in ductal carcinoma (18).

To date, autoantibodies to different classes of intermediate filaments, including vimentin, have been detected in human sera (19–22). Although the mechanism of induction of autoantibodies against vimentin still remains obscure, proteolysis of the intact, native protein may play a role in development of autoimmunity. In a previous study we have demonstrated that a particular form of calreticulin elicits a humoral response in hepatocellular carcinoma (23), with the reactive epitope occurring in a truncated form (CRT32, which includes the C-terminal portion), whereas the intact protein did not elicit reactivity. Importantly, *in vitro* studies have demonstrated that vimentin is subject to caspase-mediated proteolysis in an apoptosis-related manner (24). Prasad et al. (25) have reported apoptosis-associated proteolysis of vimentin in human prostate epithelial tumor cells and demonstrated that vimentin undergoes limited proteolysis in the apoptotic cells. Additionally, intact vimentin and some of its proteolytic fragments correspond to ubiquitinated polypeptides that are specific to the apoptotic process (26–27). Alcover et al. (27) have demonstrated that anti-vimentin antibodies in patients with autoimmune diseases interact preferentially with a specific domain of the protein, a peptide with a molecular weight of 30 kDa that is close to the amino-terminal of intact vimentin. It should be noted, however, that although we have identified 12 peptides from vimentin by MS/MS analysis, the most N-terminal of these started at residue 78 of the intact protein. Although the epitope that reacts with autoantibodies in pancreatic cancer patient’s sera remains undefined, it is plausible that the antigenic epitope was exposed following proteolytic cleavage of the intact protein near the N-terminus.

Most antigens recognized by autoantibodies are molecules that exist within cells under normal conditions. However the structure, processing and/or subcellular localization of some molecules, change with cell death. These processed forms are recognized by the immune system, leading to the development of autoimmunity particularly if the dead cells fail to be effectively cleared by phagocytosis (28). For example, some autoantigens such as the nuclear autoantigen La, or ribonucleoprotein Ro move to a region near the cell periphery during apoptosis (29–30). Thus, it may be proposed that the processing and translocation of cellular antigens to a new sub-cellular compartment could have a causative role in autoimmunity. Interestingly, it has been recently demonstrated that vimentin is secreted by macrophages in response to pro-inflammatory signaling pathways (31). In particular, whereas the anti-inflammatory cytokine interleukin-10 will inhibit vimentin secretion from macrophages, the pro-inflammatory cytokine tumor necrosis factor will trigger secretion (31). These findings may also contribute to the development of autoantibodies against vimentin in pancreatic cancer.

A prerequisite for an immune response against a cellular protein is its presentation as an antigen. It is not clear why only a subset of patients with a specific tumor type develop a humoral response to a particular antigen. Immunogenicity may depend on the level of expression, post-translational modification, or other types of processing of a protein, the extent of which may be variable among tumors of a similar type. Although vimentin expression is approximately three fold higher in pancreatic tumors at the protein level, as compared to other tumors analyzed in our study, only a single isoform of vimentin had demonstrable immunogenicity to autoantibodies in pancreatic cancer patient’s sera. Thus, the immunoreactivity of vimentin is unlikely to be related to the total level of vimentin protein expression. Further, we were unable to demonstrate aberrant N- or O-linked glycosylation of vimentin in the pancreatic tumor cell lines (data not shown).

Although the vimentin autoantibodies were largely restricted to patients with pancreatic cancer among the subject groups we investigated, further studies are needed to determine the specificity of the vimentin antibodies to pancreatic cancer. For example, although increased levels of vimentin antibodies were found in pancreatic cancer, as compared to chronic pancreatitis and healthy individuals, the relationship between tumor burden, tumor staging and antibody levels needs further clarification. Assessment of the utility of vimentin autoantibodies as diagnostic markers in pancreatic cancer also needs to be addressed in further studies. It is clear, however, that the proteomic approach we have implemented, which allows for the screening of natural forms of proteins as expressed in tumor cells, has the potential to identify novel antigens that may have utility for cancer screening and diagnosis.

## Figures and Tables

**Table 1:** Identification of vimentin autoantibodies in patient’s sera. P value is in comparison to pancreatic cancer *, one-sided Fisher’s exact test.

Serum	Number of subjects	Vimentin-positive	P value*
Pancreatic cancer	36	16 (44.4%)	
Chronic pancreatitis	18	1 (5.6%)	P = 0.003
Healthy Individuals	15	0 (0%)	P = 0.001

**Table 2:** Expression levels of the antigenic vimentin isoform in human tumors. The numbers shown are averaged integrated intensity measurements (± SD) for two vimentin spots, with “A” being the ubiquitous spot and “B” being the identified antigenic spot.

Tumor Type	Gels Analyzed	Spot A measurement	Spot B measurement	B/A
**Colon**	18	0.668 ± 0.255	0.574 ± 0.21	0.859
**Ovarian**	16	0.284 ± 0.173	0.176 ± 0.086	0.620
**Lung**	14	0.359 ± 0.317	0.450 ± 0.205	1.253
**Esophageal Pancreas**	10	0.816 ± 0.33	0.861 ± 0.277	1.055
**Normal Pancreas**	4	0.471 ± 0.264	1.911 ± 0.263	4.057
**Tumor**	4	0.215 ± 0.119	1.369 ± 1.334	6.367
